# Stable Decoding from a Speech BCI Enables Control for an Individual with ALS without Recalibration for 3 Months

**DOI:** 10.1002/advs.202304853

**Published:** 2023-10-24

**Authors:** Shiyu Luo, Miguel Angrick, Christopher Coogan, Daniel N. Candrea, Kimberley Wyse‐Sookoo, Samyak Shah, Qinwan Rabbani, Griffin W. Milsap, Alexander R. Weiss, William S. Anderson, Donna C. Tippett, Nicholas J. Maragakis, Lora L. Clawson, Mariska J. Vansteensel, Brock A. Wester, Francesco V. Tenore, Hynek Hermansky, Matthew S. Fifer, Nick F. Ramsey, Nathan E. Crone

**Affiliations:** ^1^ Department of Biomedical Engineering Johns Hopkins University School of Medicine Baltimore MD 21205 USA; ^2^ Department of Neurology Johns Hopkins University School of Medicine Baltimore MD 21287 USA; ^3^ Department of Electrical and Computer Engineering Johns Hopkins University Baltimore MD 21218 USA; ^4^ Center for Language and Speech Processing Johns Hopkins University Baltimore MD 21218 USA; ^5^ Research and Exploratory Development Department Johns Hopkins University Applied Physics Laboratory Laurel MD 20723 USA; ^6^ Department of Neurosurgery Johns Hopkins University School of Medicine Baltimore MD 21205 USA; ^7^ Department of Otolaryngology‐Head and Neck Surgery Johns Hopkins University School of Medicine Baltimore MD 21205 USA; ^8^ Department of Physical Medicine and Rehabilitation Johns Hopkins University School of Medicine Baltimore MD 21205 USA; ^9^ Department of Neurology and Neurosurgery UMC Utrecht Brain Center Utrecht 3584 The Netherlands

**Keywords:** amyotrophic lateral sclerosis (ALS), brain‐computer interfaces, neural decoding, speech brain‐computer interface (BCI)

## Abstract

Brain‐computer interfaces (BCIs) can be used to control assistive devices by patients with neurological disorders like amyotrophic lateral sclerosis (ALS) that limit speech and movement. For assistive control, it is desirable for BCI systems to be accurate and reliable, preferably with minimal setup time. In this study, a participant with severe dysarthria due to ALS operates computer applications with six intuitive speech commands via a chronic electrocorticographic (ECoG) implant over the ventral sensorimotor cortex. Speech commands are accurately detected and decoded (median accuracy: 90.59%) throughout a 3‐month study period without model retraining or recalibration. Use of the BCI does not require exogenous timing cues, enabling the participant to issue self‐paced commands at will. These results demonstrate that a chronically implanted ECoG‐based speech BCI can reliably control assistive devices over long time periods with only initial model training and calibration, supporting the feasibility of unassisted home use.

## Graphical abstract

A speech brain‐computer interface enables a clinical‐trial participant with amyotrophic lateral sclerosis to control a communication board and home devices without model retraining or baseline calibration. Six intuitive speech commands are accurately detected and decoded (median accuracy: 90.59%) from a chronic electrocorticographic (ECoG) implant over the ventral sensorimotor cortex throughout a 3‐month study period.

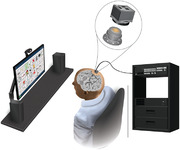

## 1 Introduction

Brain‐computer interfaces (BCIs) enable the control of devices and software applications by interpreting the user's intent from recorded brain signals.^[^ ^1^, ^2^ ^]^ BCIs may be used by individuals with severe paralysis to complement or replace their existing communication abilities and/or to control devices in their environment.^[^ ^3^, ^4^ ^]^ Under these circumstances, ideally a BCI user can issue commands whenever needed, without external cueing or assistance, and with high confidence that the command will be executed.^[^ ^5^ ^]^

Recently, there has been a dramatic emergence of speech BCI capabilities, with audio waveforms and entire sentences translated directly from intracranial recordings.^[^ ^6^, ^7^, ^8^, ^9^, ^10^, ^11^, ^12^, ^13^, ^14^ ^]^ These techniques offer a promising path toward restoring communication, but are not intended for device control, a crucial need among individuals who are severely paralyzed.^[^ ^15^ ^]^ Unlike communication BCIs, BCIs for device control must provide low‐latency, reliable commands with minimal linguistic context or assistance from language models. Moreover, the use of BCIs to interface with physical devices (e.g., thermostats, robotic assistants) arguably requires even higher decoding performance and stability over time for safety reasons.

Implantable BCIs have enabled increasingly sophisticated functionality to users, but it is challenging to maintain high performance over long periods of time without the need to retrain or recalibrate, which can be time‐consuming and require intervention by a research team or a caregiver.^[^ ^4^, ^16^, ^17^, ^18^, ^19^ ^]^ One study has demonstrated that time spent recalibrating can be reduced to as little as 2 min for baseline collection in a cursor‐control task, proving the feasibility of a ‘plug‐and‐play’ BCI.^[^ ^20^ ^]^ However, it is not clear if long‐term decoding without retraining and recalibration is still feasible for speech BCI. It also remains unknown whether daily baseline data collection, as was necessary for the aforementioned study, can be eliminated altogether, which would give more autonomy to BCI users and allow for broader BCI usage.

Here, we present a ‘plug‐and‐play’ BCI control system based on real‐time decoding of speech‐related neural activity from a chronic electrocorticographic (ECoG) implant. Using this BCI system, a study participant with amyotrophic lateral sclerosis (ALS) was able to freely generate a set of control commands (i.e., up, down, left, right, enter, and back). Commands were reliably detected and decoded as the participant navigated a communication board and controlled devices like room lights and streaming TV applications. We found that decoder retraining and recalibration, as well as baseline collection before each session, were not required to sustain high performance over a 3‐month study period following initial laboratory calibration. Together, these results provide evidence that an implanted BCI system decoding speech commands can enable a reliable and stable means of controlling a computer and other external devices over a period of several months for individuals with motor speech impairments due to neurological disorders such as ALS.

## 2 Results

### 2.1 Real‐Time Neural Decoding

**Figure 1:**
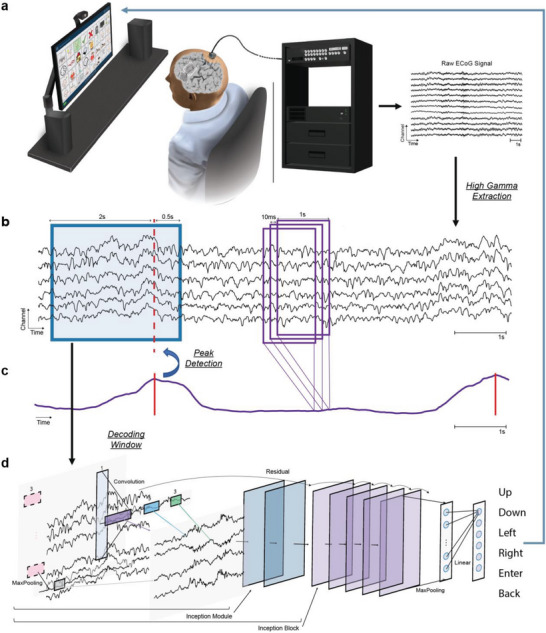
Schematics of the speech BCI for functional control. a) Neural signals were acquired from two 64‐channel ECoG arrays implanted over the motor and somatosensory areas responsible for upper extremity and speech functions. Only the inferior array was used in this study. b) A sample of high gamma energy (HGE, 70–170 Hz, z‐scored) for six channels. c) 1‐s rolling average of channel‐averaged HGE (updated every 10 ms). The peak of this signal was used to detect speech intent. Once the intended speech was detected, a decoding window consisting of HGE 2 s before and 0.5 s after the peak was sent to the classifier. d) The CNN model (InceptionTime^[^ ^25^ ^]^) classified the window of HGE into commands that facilitated navigation of a communication board or control of external devices.

A participant in the CortiCom clinical trial (ClinicalTrials.gov Identifier: NCT03567213; see Experimental Section for details) with severe dysarthria due to ALS was able to control external devices (Video S1, Supporting Information, the participant gave informed consent for the release of video recordings) and navigate a 4 × 8 communication board (**Figure** **1a**; Video S2, Supporting Information) using a BCI in real‐time. No device‐related adverse events or serious adverse events have occurred to date. The investigational BCI device is still operating nominally at the time of the reporting, acquiring good‐quality ECoG signals in all but four of the 128 ECoG electrodes. Two 64‐channel high‐density ECoG arrays were implanted over motor and somatosensory cortical areas (Figures 1a and 4a). The lateral array primarily covered brain regions responsible for speech‐related functions; this array alone was used to decode speech commands in this study. Raw ECoG signals were bandpass filtered between 70 and 170 Hz to estimate high gamma energy (HGE), which has been shown to correlate with neuronal population activity underlying the electrodes^[^ ^21^, ^22^, ^23^ ^]^ and used widely to decode speech from ECoG signals.^[^ ^8^, ^9^, ^24^ ^]^ The event‐related increases in HGE were used by the BCI system to determine whether a command had been issued by the user (Figure 1b,c). Specifically, the time associated with the local maximum of a 1‐s rolling average of channel‐averaged HGE was identified. Once a speech event was detected, we classified neural features in a window consisting of HGE 2 s prior to peak detection and 0.5 s after peak detection using a convolutional neural network (CNN, InceptionTime,^[^ ^25^ ^]^ Figure 1d). Visual feedback was presented to the participant as soon as decoding results were received by the system. In the communication board navigation task, the participant could see a red highlight moving in cardinal directions when the decoding results were up, down, left, or right, respectively. This red highlight turned green if the command enter was decoded. When a back command was decoded, a yellow highlight replaced any existing highlight, which indicated that the current option had been unselected.

The CNN decoding model was trained on data collected during a word production task where the subject was instructed to read the six commands as they appeared on the screen. Training data collection for this task began and concluded 77 and 120 days post‐implant (4 and 3 months prior to real‐time usage), respectively. To accommodate for the usage of the system without recalibration, all data were normalized using the mean and standard deviation of the silence period (0.8 to 0 s prior to stimulus onset) in a syllable repetition task (Note S1, Supporting Information) collected from a single, arbitrarily chosen day (95 days post‐implant) in the aforementioned time frame.

### 2.2 Stable Decoder Performance Over Three Months

Real‐time testing began 194 days after implantation, at which point all model parameters were fixed and no retraining of the decoding model took place. On each day of testing, the participant was instructed to issue verbal commands (up, down, left, right, enter, and back) at his own pace to navigate across the communication board toward targets of his choice. HGE features were normalized with statistics collected on Day 95 post‐implant. No separate baseline collection/model recalibration took place prior to real‐time experiments. We report here performance statistics in a 3‐month study period, concluding 285 days post‐implant (n = 35 sessions in total). For the first 2 days of real‐time usage, two sessions were conducted each day. All other days had a single session lasting just under 5 min on average (Table S1, Supporting Information).

**Figure 2:**
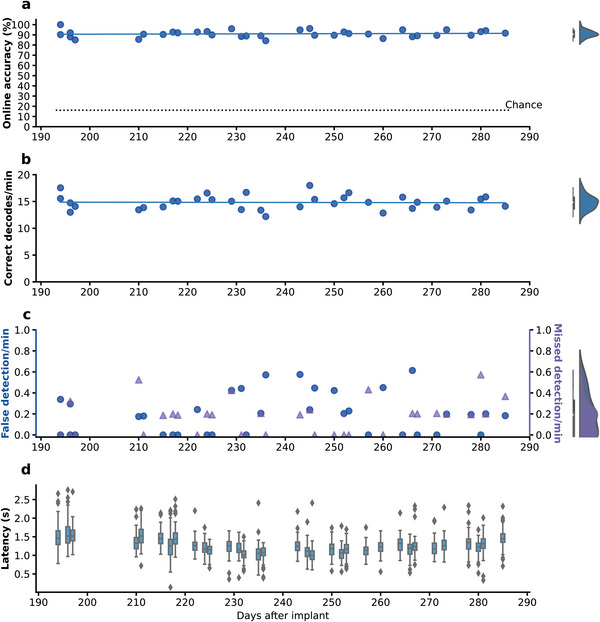
Stable performance of the BCI in online self‐paced experiments over 3 months. a) Online accuracy of the BCI system. Each dot represents one session. Average chance = 16.16% (n = 10000 simulations, dashed line). The blue line is the linear least squares regression line between accuracy and days after implant. b) Correct decoding results performed by the BCI per minute. Each dot represents one session. The blue line is the linear least squares regression line between correct decodes per minute and days after implant. c) Number of false detections (blue dot) and missed detections (purple triangle) per minute. Each symbol represents one experiment session. d) Time between speech offset and when the decoding result was registered by the BCI system for every successful decode per day. For all boxplots, the center line represents the median, top and bottom edges of the box represent quantiles. Data outside of 1.5 times of interquartile range were shown as outlier data points, and the maximum and minimum of non‐outliers were shown as whiskers.

As all commands were selected by the participant overtly in real time, audio recordings of the participant during online usage were transcribed as ground truth. We define online accuracy as the percentage of real‐time classification results matching the transcriptions when a command was indeed issued by the participant. The participant achieved a median accuracy of 90.59% (95% CI: [89.47%, 92.00%], **Figure** **2**a). Performance held steady across the study span, with no significant relationship between online accuracy and days after implant (y = 0.010x + 88.70, where x is the number of days after implant (same below), R^2^ = 0.006, p = 0.65, Figure 2a). The median correct decodes per minute across sessions was 14.9 (95% CI: [14.0, 15.3], Figure 2b, the median for commands issued per minute was 16.49), and its relationship with days after implant also could not be established (y = −0.001x + 15.11, R^2^ = 0.001, p = 0.88, Figure 2b). These findings indicate the stability of our decoder even without retraining or dedicated baseline recalibration.

Separately, we measured the performance metrics of our detection algorithm (Figure 2c). False detections were defined as speech events detected by the algorithm while the participant did not attempt to say a word. Missed detections were defined as instances where the participant finished a full word that the detection algorithm failed to detect. Across the study period, both the false detection rate and missed detection rate remained low. The median false detection rate was 0.19 min^−1^(95% CI [0.00, 0.23], y = 0.001x − 0.027, R^2^ = 0.017, p = 0.45), and the median missed detection rate was 0.19 min^−1^ (95% CI [0.00, 0.20], y = 0.002x − 0.208, R^2^ = 0.066, p = 0.14). We found no statistically significant linear trend between either of these metrics and days after implantation. Additionally, the median time interval between speech offset and when the decoding results were registered by the BCI system was 1.24 s (95% CI: [1.23, 1.25], Figure 2d). This response speed represented the delay of the system between when the participant issued the instruction and when the system completed the corresponding action.

### 2.3 Stability of the Decoding Signals

**Figure 3:**
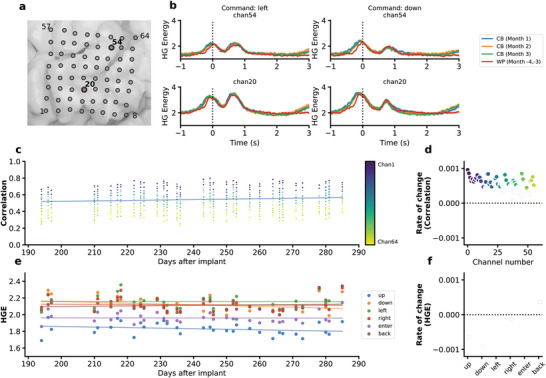
Stability of the event‐related high gamma activities acquired from the ECoG arrays. a) Anatomical location of the ECoG array used in this study. Example channels in (b) are highlighted. b) Examples of event‐related HGE in both training and real‐time usage phases for two different commands. A vertical dotted line at 0 s indicates speech onset. The shaded area represents 95% CI. CB: Communication Board (real‐time usage). WP: Word Production (training data). c) Correlation between the real‐time usage trials and average training data per channel. For each real‐time usage trial, the Pearson's correlation coefficient between its HGE and the average HGE of the corresponding command in training data collection phase was calculated. Each dot represents the average (weighted against the frequency of the command) of the correlation coefficients per usage day per channel. The blue line represents the linear least squares regression line between channel‐averaged correlation and days after implant. d) Rate of change for correlation. Each dot represents one channel. Filled dots represent statistically significant linear relationships between correlation values and days after implant (p <0.05, Wald test with t‐distribution). Unfilled dots indicate that a relationship could not be established (p > = 0.05). e) Channel‐average of logarithmic HGE (unnormalized) for each command during online usage. Lines represent the linear least squares regression lines between HGE and days after implant for each command. f) Same as (d), but for HGE.

To quantify the stability of the underlying neural signals, we investigated both the neural features used for the training of the decoding model and those collected during the real‐time testing phase. **Figure** **3**b displays the HGE over a time period of 4 s, starting from 1 s prior to speech onset, for two example electrodes (location shown in Figure 3a). Here, HGE is aggregated by month when using the communication board online, or from the collection of training data—colorized for each case differently. We saw similar patterns of event‐related HGE increase during the training data collection phase and each month that the BCI system was used in real‐time (Figure 3b; Figures S5 and S6, Supporting Information).

We then compared the similarity between the time series (−1.0 to 1.5 s relative to speech onset) of raw event‐related HGE during the model training phase and each day of real‐time usage. Figure 3c reports Pearson's correlation coefficients for each channel across days. Despite the variance of correlation values across channels, the correlation pattern between neural activity during training and real‐time use was relatively stable. A small increasing trend was observed for the channel average (y = 0.001x + 0.410, R^2^ = 0.183, p <0.05, Figure 3c). For n = 37/60 channels, a small (slope <0.001 day^−1^) but statistically significant (p <0.05) increase in correlation scores over time was observed (Figure 3d). For n = 23/60 channels, no significant relationship between correlation coefficients and days after implant could be established (Figure 3d). These findings suggest that the neural signals maintained their relative similarity to the training data during the course of real‐time usage.

Lastly, we monitored the stability of the neural signals from the perspective of raw HGE. We computed the average HGE across channels for each command (−1.0 to 1.5 s relative to speech onset) during each day of online usage (Figure 3e). We observed no statistically significant (p < 0.05) relationship between command‐specific HGE and days after implant (Figure 3f).

### 2.4 Electrode Contribution

**Figure 4:**
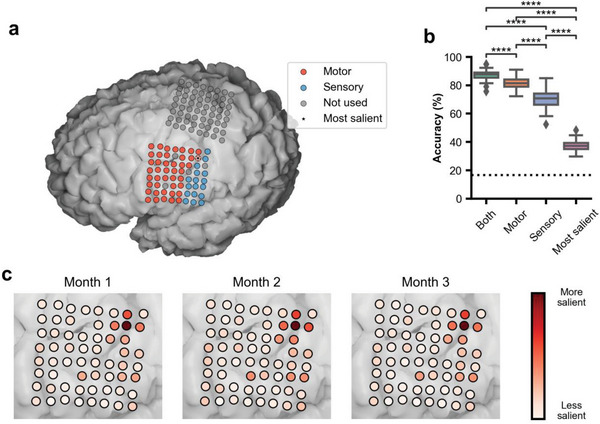
Electrode contribution during the study period. a) MRI reconstruction of the participant's brain, overlaid on top of which are the ECoG grids implanted as part of the clinical trial. Electrodes used in this study are colored in red (motor) and blue (sensory). The grey electrodes were not used in this study. b) Simulated online accuracy when the decoding model is trained with both motor and sensory electrodes, only motor electrodes, only sensory electrodes, and only the most salient electrode. Chance = 16.67% (shown as dashed line). Each box corresponds to the accuracy for n = 33 testing days (^****^p < 0.0001, Mann–Whitney‐Wilcoxon test two‐sided with Bonferroni correction). c) Relative contribution of each of the electrodes to the decoding results for each real‐time usage month.

Next, we examined that electrodes among those selected made the strongest contribution to decoding performance and stability. We first tested whether similar decoding performance could have been achieved had the ECoG grid only covered either motor (including premotor) or sensory cortices (pre‐versus post‐central areas). We simulated real‐time usage of a motor‐only and a sensory‐only model using neural activity data from online sessions, detected from the aforementioned methods using all 60 electrodes. Lower accuracies were observed in both motor‐only (median: 81.33%, 95% CI [79.07%, 83.33%]) and sensory‐only (median: 70.67%, 95% CI [66.67%, 73.49%]) conditions (**Figure** **4**b, p < 0.0001, Mann–Whitney‐Wilcoxon test, two‐sided with Bonferroni correction for six comparisons). As with the full model, no statistically significant trend between decoding accuracies and days from implantation could be established (y_1_ = −0.058 x + 95.186, R^2^ = 0.109, p = 0.06; y_2_ = −0.016 + 74.159, R^2^ = 0.004, p = 0.73 where y_1_ and y_2_ are accuracies with models trained with sensory and motor electrodes excluded, respectively; Figure S2, Supporting Information). These findings suggest that the wide coverage of the ECoG grid might have been necessary to achieve the high performance we observed, though performance stability did not seem to be affected by reduced coverage.

We then took a more granular look at which specific electrodes had the greatest influence on decoding. Each electrode's influence on decoding was quantified as relative changes to model predictions based on small perturbations to the neural activity from that electrode.^[^ ^26^ ^]^ The dorsal and posterior parts of the grid had more influence on the decoding model than the ventral and anterior parts of the grid. The most influential electrodes were localized to the dorsal part of the ventral sensorimotor cortex (vSMC), which has been associated with lip movement, and to a lesser extent, to areas associated with tongue and jaw movement.^[^ ^27^ ^]^ We then investigated whether this spatial pattern of electrode influence was stable across the study period (Figure 4c). Strong correlations (Pearson's correlation coefficient, r = 0.985 between 1 and 2 months, r = 0.994 between 1 and 3 months, r = 0.992 between 2 and 3 months, p < 0.0001 for all three pairs) between the influence of the electrodes across the three real‐time usage months were observed.

### 2.5 Generalizing Stable Performance to Functional Control and Silent Speech

**Figure 5:**
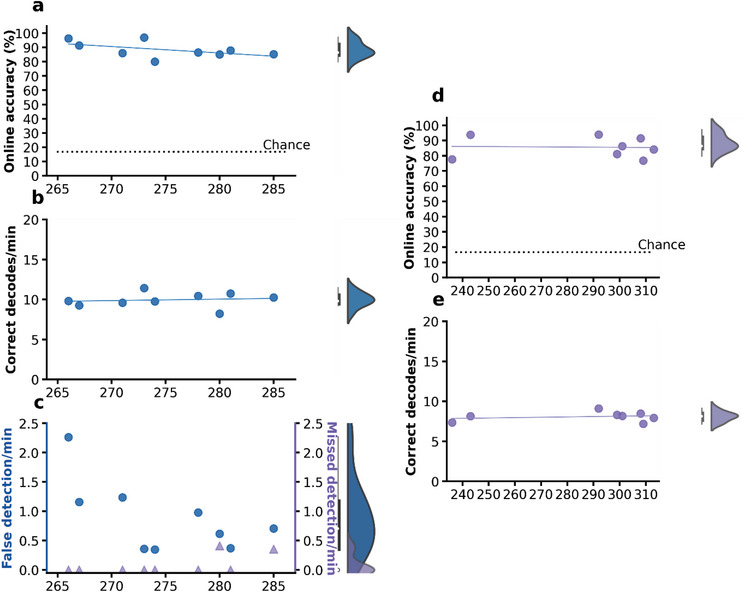
Performance in functional control and during silent (mimed) speech. a) Online accuracy of the BCI system during functional control. Each data point represents one session. Chance = 16.67% (dashed line). The blue line is the linear least squares regression line between accuracy and days after implant. b) Correct decoding results performed by the BCI per minute during functional control. Each dot represents one session. The blue line is the linear least squares regression line between correct decodes per minute and days after implant. c) Number of false detections (blue dots) and missed detections (purple triangles) per minute during function control. Each symbol represents one session. d) Online accuracy of silent speech decoder. Each dot represents one day. Average chance = 16.73% (dashed line, n = 10000 simulations). The purple line represents the linear least squares regression between accuracy and days after implant. e) Correct decoding results performed by the BCI per minute using the silent speech decoder. Each dot represents one day. The purple line is the linear least squares regression line between correct decodes per minute and days after implant.

Since many aspects of the decoding system design were motivated by the need for ALS patients to interface with computational devices and external hardware, we examined whether the stability and performance of a fixed decoder could still hold under hardware‐control conditions. In these functional control experiments, options on the communication board were linked with real‐world events (e.g., turning a light on and off, contacting caregivers, playing the radio, etc.). The participant was also given the ability to control a TV application on a separate screen (Video S1, Supporting Information), which also consisted of 2‐D arrays of menu options that were navigated using the same six commands. Online functional control experiments started 266 days post‐implant and concluded 285 days after implant (coinciding with the last month of online communication board usage experiments). The same fixed decoder as described above was used without any further training or recalibration. The participant was instructed to finish a series of functional tasks at his own pace in the following order: activate the BCI system (by issuing three enter commands in a row), turn off a lamp, turn on the radio, turn off the radio, activate smart TV control, open a video application, and select a video to watch. Median accuracy across the nine functional control sessions was 86.49% (95% CI: [80.00%, 96.30%]). No statistically significant relationship between functional control accuracy and days after implant could be established (y = −0.450 x + 212.21, R^2^ = 0.278, p = 0.15, **Figure** **5**a). The median for correct decodes per minute was 9.8 (95% CI [8.2, 10.7]), with no significant relationship with days after implant (y = 0.018 x + 4.975, R^2^ = 0.016, p = 0.74, Figure 5b). Note that this number was substantially lower than the correct decodes per minute reported earlier for communication board control. This was mainly because the participant required more time to examine their options, and to a lesser extent, wait for control commands to take effect in the real world. The median false detection rate was 0.71 min^−1^(95% CI [0.35, 1.24]), and median missed detection rate was 0 min^−1^ (95% CI [0.00, 0.35]). No statistically significant relationship between either metric and days after implant could be established (y_1_ = −0.061 x + 17.543, R^2^ = 0.405, p = 0.06; y_2_ = 0.017 x − 4.494, R^2^ = 0.406, p = 0.06, where y_1_ is false detection rate and y_2_ missed detection rate, Figure 5c). Together, these findings suggest that the performance and stability of our BCI system also apply to functional control under simulated real‐life settings in laboratory.

We then examined whether stable performance could be achieved if no audible speech was produced. A separate decoder was trained using data collected in a word production task where the participant silently mimed the words shown on the screen. Data collection for the training of the silent speech decoder started and concluded 83 and 225 days after implant, respectively, across eight different days. The participant was instructed to silently move his articulators and facial muscles in these sessions; all other parameters of the task paradigm were kept consistent with the aforementioned word production task. Online usage of this new decoder started and concluded 236 and 313 days after implant, respectively. In these experiments, a cross occurred on the screen for 2 s prompting the participant to give a silent command, chosen freely by him. The decoding window started when the cross disappeared and ended 2.5 s after. Once the cross disappeared, the user interface displayed the same 4 × 8 communication board as referenced above in the real‐time communication board usage experiments. The participant was instructed to issue commands once the cross disappeared. The lip movements of the participant were transcribed as ground truth. Once the decoding result was received by the user interface, the highlight on the communication board changed accordingly and the results were presented to the subject for a further 1.5 s before the next cross replaced the communication board display. Median accuracy across the eight silent speech control sessions was 85.18% (95% CI: [76.79%, 93.75%]). No linear trend of decoding accuracy could be established across days after implant (y = −0.010 x + 88.437, R^2^ = 0.002, p = 0.92, Figure 5d). The median for the number of correct decodes per minute was 8.16 (95% CI [7.2, 8.5], Figure 5e). These results indicate that BCI stability can also be achieved in the absence of phonation.

## 3 Discussion

In this study, we demonstrated the accuracy and stability of a speech BCI system based on a chronic ECoG implant. No serious adverse events or device‐related adverse events occurred during the study period. At the time of this report, the BCI system is still operating nominally, capable of streaming all but 4 of 128 ECoG signals. A clinical‐trial participant was able to use the system at his own pace to control computer applications and external devices over a 3‐month study period without model recalibration or retraining. Previous demonstrations of implanted speech BCIs have focused primarily on the restoration of communication abilities for participants by translating brain activity into text.^[^ ^24^, ^28^ ^]^ An outstanding question is whether decoding neural activities during speech commands can also be used to enable the direct control of devices, another crucial need among severely paralyzed individuals. In a control‐oriented BCI, similar to voice assistants used by able‐bodied individuals, each user‐issued command needs to be detected and classified with high confidence. Otherwise, BCI users may elect to abandon device use.^[^ ^29^ ^]^ Here, we show that a speech BCI using a small set of intuitive commands can indeed achieve a high accuracy for performant navigation of a communication board, as well as to control household devices, without the use of a language model to correct for decoding errors.

Another outstanding question for BCI development is whether day‐to‐day setup time associated with retraining and baseline collection can be reduced while still maintaining robust performance.^[^ ^30^ ^]^ This problem becomes more acute for control‐focused BCIs, as their ultimate purpose is to facilitate the independent use of assistive and other smart devices at home. Individuals with severe motor impairments need to be able to reliably control BCI systems whenever the need arises. Previous studies have already demonstrated that the neural representations responsible for consistent motor behaviors that underlie BCI control are stable.^[^ ^31^, ^32^ ^]^ A few recent studies have reduced the setup time for BCI systems to as little as 2 min.^[^ ^18^, ^20^ ^]^ Our study builds on these prior works by eliminating the model retraining and baseline recalibration steps altogether, marking a critical step toward the independent usage of a speech BCI for navigational control without the need for ongoing researcher intervention.

What drove the decoding stability in our study? We believe the stability of high gamma responses recorded from the implanted ECoG arrays had an important impact on decoding stability. Previous studies have demonstrated short‐ and long‐term stability of ECoG implants in both humans^[^ ^3^, ^20^, ^33^, ^34^, ^35^ ^]^ and non‐human primates.^[^ ^36^, ^37^ ^]^ In this study, we extended these findings on chronic ECoG decoding stability to speech BCI in an ALS participant. Our results add to the growing evidence that high gamma responses are highly informative for speech‐related motor behaviors,^[^ ^7^, ^20^, ^21^, ^38^ ^]^ and that they are stable, likely because they reflect the collective firing rates of neuronal populations.^[^ ^21^, ^22^, ^23^ ^]^ Further studies are needed to explore in detail the stability of these and other spectral features of ECoG signals.

One limitation of our approach was the limited vocabulary used for speech decoding. Although the six commands we adopted in this study were both intuitive and sufficient for controlling grid‐based applications, a more comprehensive vocabulary may reduce the time needed to perform each selection. Previous reports on speech BCIs have demonstrated that a higher number of decoding classes can still be accurately decoded.^[^ ^28^ ^]^ The success of speech synthesis directly from neural recording also suggests the potential for decoding a larger vocabulary.^[^ ^6^, ^8^, ^9^ ^]^ The current study was conducted with only a single participant in a phase I clinical trial of the safety and feasibility of an ECoG‐based BCI device. Few studies have explicitly tested the effectiveness of an ECoG‐based speech BCI in people living with ALS.^[^ ^3^ ^]^ Further studies are needed to verify if our proposed approach will generalize to other participants with similar conditions. Even though we explored how various other popular BCI approaches would perform in offline simulations, we did not have the opportunity to establish how those approaches would perform in the same online close‐loop experiments for longer periods of time. For example, the addition of lower‐frequency information or analytic amplitude extraction of high gamma could have potentially resulted in better online decoding accuracy. Our participant had severe dysarthria due to bulbar dysfunction, with limited intelligibility. Nevertheless, he was still capable of phonation and articulation. Even though we were able to achieve high performance with silent speech, it still remains to be seen whether the same level of performance can be achieved in people living with ALS who are unable to phonate and/or articulate.

## 4 Conclusion

Overall, our work demonstrates the potential of safely using implanted BCIs for intuitive control of external devices for a prolonged time. In future studies, it might be possible to extend the capability of decoding systems that require no recalibration to longer and more diverse voice commands. In a home‐use BCI system, stable decoders such as ours can also be used to initiate calibration sessions for more sophisticated, albeit less stable, decoders. Utilizing the signal stability afforded by intracortical ECoG recordings, our results may be one of the first steps in realizing the potential for independent home use of speech BCIs by individuals with severe paralysis.

## 5 Experimental Section

### Clinical Trial and Participant

This study included data from one clinical trial participant (ClinicalTrials.gov Identifier: NCT03567213) who gave written informed consent for implantation of the study device and all research activities associated with this study. The use of the device in this study was approved by the US Food and Drug Administration under an Investigational Device Exemption. The study protocol was approved by the Johns Hopkins Medicine Institutional Review Board (IRB00167247). The participant was a right‐handed man who was 61 years old at the time of implant. He was diagnosed with ALS ≈8 years prior to the start of the study. The participant had severe progressive dysarthria and dysphagia as a result of bulbar dysfunction. This had also been accompanied by progressive dyspnea. His residual phonation and articulation ability could still support overt speech, albeit with reduced speed and limited intelligibility. In addition, he had experienced progressive upper limb weakness, but his lower limbs were less affected.

### Safety and Signal Monitoring

The primary outcome measures of the clinical trial described here were designed to establish the safety and recording viability of the investigational BCI device. The safety outcome was measured by time to device explanation and was considered a success if the device was not explanted due to safety concerns during the study period. At the time of this reporting, the device was implanted for 52 weeks. Safety monitoring of the device throughout the study period had consisted of visual inspections of the device, review of streamed ECoG signals, assessments of vital signs, mood, and cognition at every study visit, as well as monthly physical, neurological, and cognitive examinations. No serious adverse events or device‐related adverse events had been reported to date. The recording viability of the study device was measured as the number of usable neural signals. All but 4 of the 128 ECoG electrodes had produced usable neural signals throughout the study duration to date. Three electrodes with consistently high impedance (>15kΩ) were excluded from the analysis. Impedances for the rest of the electrodes remained under 15kΩ throughout the study period. One additional electrode was excluded due to inconsistent signal quality confirmed with visual inspection of the raw signals. On a few occasions, raw signal recorded from that channel did not exhibit nominal patterns of brain waves and remained lower in amplitude than normal. ECoG signals from the remaining 60 electrodes of the ECoG array implanted over ventral sensorimotor cortex were used for training and testing decoding algorithms.

### Surgical Implantation

Implantation of the investigational device occurred in July 2022 at the Johns Hopkins Hospital without surgical complications. Two 64‐channel ECoG grids (PMT Corporation, Chanhassen, MN) were implanted subdurally on the pial surface of the brain, over brain regions responsible for speech and upper extremity movements. Exact placement was informed by anatomical landmarks, preoperative fMRI, and somatosensory evoked potentials. Each ECoG grid had a surface area of 12.11 cm^2^ (36.66 mm x 33.1 mm) with an 8 × 8 electrode configuration and 4 mm center‐to‐center spacing embedded in soft silastic sheets. Each platinum‐iridium disc electrode had a thickness of 0.76 mm and an exposed surface diameter of 2 mm. Two wires implanted over the surface of the grids served as a reference for ECoG signal amplification. One percutaneous pedestal connector (Blackrock Microsystems, Salt Lake City, UT) connected to the ECoG grids was surgically anchored to the skull.

### Activity Detection

The channel average of the normalized high gamma signal of the 60 included electrodes across a 1‐s integral window was calculated every 10 ms and used for detection of neural activity associated with speech (referred to as detection signal henceforth, Figure 1b,c). The peak from a 3‐s rolling buffer of the detection signal was identified if its prominence^[^ ^39^ ^]^ was over two times the standard deviation of a 10‐s rolling window of detection signals. A 2.5‐s window (2 s before the peak and 0.5 s after the peak) of the processed neural activity was then used for the classification of the six speech commands (neural decoding model, Figure 1b). The same detection algorithm was also used for extracting training samples.

### Neural Decoding Model

For the neural decoding model, a convolutional neural network (CNN) was designed using the InceptionTime architecture.^[^ ^25^ ^]^ Targeted toward time series classification, the InceptionTime model incorporated filters of variable length to gain access to hierarchical latent structures of different time resolutions. In the implementation of the CNN, Six Inception^[^ ^40^ ^]^ blocks were used, each with three Inception modules (Figure 1d), without neural network ensembling. Inside each Inception module, three sets of convolutions, each with 32 filters with kernel sizes ∈ {5, 11, 23}, were used after an initial convolution layer with 32 filters of kernel size = 1. A MaxPooling layer with kernel size = 3 and one subsequent set of convolutions with 32 filters of kernel size = 1 were also used within each module. The output of the four sets of convolutions was concatenated to form the output of each module. The final output from the last Inception block was used as input to a MaxPooling layer, followed by a fully connected layer that provided the final predicted classification score. The model in Python 3.8/3.9 was implemented using PyTorch v1.10. See Note S2 (Supporting Information) for details on the decoding model.

### Data Collection and Model Training

The overt speech command decoding model was trained on data collected between Day 77 and Day 120 post‐implant. The silent speech decoding model was trained on data collected between Day 83 and Day 225 post‐implant. The subject was instructed to read aloud or silently mime single text commands as each appeared on a computer monitor (Note S1, Supporting Information). There were in total 30 data collection experiments for overt speech conducted across 11 days, totaling 142.8 min and 300 trials for each command. Data collection experiments (43 in total) were conducted for silent speech across 17 days, totaling 266.6 min and 430 trials for each command. Decoding model optimization was performed with Adam optimizer.^[^ ^41^ ^]^ Model performances were evaluated with different hyperparameter choices by withholding an entire day's data as validation set.

### Data Preprocessing and Real‐Time System

ECoG signals were filtered (0.3–7500 Hz), amplified, and digitized using a NeuroPlexE headstage (Blackrock Microsystems, Salt Lake City, UT) attached to the 128‐channel percutaneous connector of the implanted investigational device. The headstage was connected by HDMI cable to a Digital NeuroPort Biopotential Signal Processing System (NSP, Blackrock Microsystems, Salt Lake City, UT) where signals were downsampled to 1000 Hz. During real‐time usage, data from the NSP was streamed from a ZeroMQ^[^ ^42^ ^]^ server implemented in the signal processing module of BCI2000.^[^ ^43^ ^]^ Subsequent real‐time signal processing and model inference were implemented in Python within the ezmsg framework, a directed acyclic messaging pattern (https://github.com/iscoe/ezmsg), and deployed on a desktop computer with an Ethernet cable connection to the host computer for the Neuroport System. A ZeroMQ subscriber within this framework received and reformatted the data. Subsequently, data from the 60 channels used in this study were selected. High gamma signals were extracted using an 8th order Butterworth bandpass filter between 70 and 170 Hz. A notch filter between 118 and 122 Hz was applied to remove line noise. The logarithmic power of the high gamma signal for a 50‐ms window was computed once 10‐ms data was received. These processed HGE features were then stored in a decoding data buffer pending the detection of speech intent.

The channel average of HGE was stored in a separate 1‐s circular buffer. Every 10 ms, the time average of the circular buffer was computed and stored in a detection buffer that was 3 s in duration. A 10‐s baseline buffer was also updated the same way. Any local maximum in the detection window whose prominence was over two times the standard deviation of the detection window was defined as a detection. Peak detection was rejected if the difference between the signal peak and the minimum of the baseline buffer was below the standard deviation of the baseline buffer. Otherwise, the detected peak time was sent to the decoding data buffer.

Once the detected peak time was received by the decoding data buffer, another 0.5‐s worth of data was collected. This was appended to the 2 s of data prior to peak detection. This 2.5‐s window of data was then normalized against the mean and standard deviation of the pre‐trial silent period (0.8 to 0 s prior to stimulus onset) collected during a syllable repetition task conducted 95 days after implant. Next, the fixed CNN decoder classified these neural signals into commands. Decoding results with raw scores at or above 0.55 were considered valid results and were sent back to the host machine. Results with raw scores below 0.55 were not registered and were treated as missed detections if a speech attempt was made.

In the real‐time communication board control tasks, for ease of use, a command after enter would only be considered valid if it were back. In the case of any other commands being decoded, the application considered them to be back. In functional control tasks, this feature was not enabled as directional commands were still valid options after enter.

### Online Experimental Task Design

In the real‐time communication board control task, the subject was asked to freely choose his own target on a 4 × 8 communication board and navigate toward the target by issuing verbal commands. The application started with a red highlight over one of the icons on the communication board. This highlight turned green if the enter command was received, and would move rightward, leftward, upward, or downward if the right, left, up, or down command was received, respectively. The highlight turned yellow upon receiving the back command.

In the real‐time functional control task, operation of the application started with the participant saying three enter commands in a row. The participant was asked to navigate in an application displayed on the screen directly in front of him. This application had an identical layout to the aforementioned communication board but provided different options. A separate screen displaying a streaming TV application was placed to the front left of the participant. The TV application was put into standby mode at the start of the experiment. Once the experiment started, the participant was instructed to first enter the functional control application. He was asked to then navigate to the correct icons by issuing up, down, left, or right commands. After the correct icon was selected, an enter command triggered functional control associated with the selected icon. If the TV application was selected, all further commands were then relayed to the TV application in lieu of the functional control application.

In the silent speech control task, a cross occurred on the screen for 2 s prompting the participant to give the next command. At the end of the 2 s, the cross was replaced by the communication board application. The participant was instructed to silently mime a freely chosen command immediately after the cross disappeared. Once the decoding result was received by the application, the highlight on the communication board was changed accordingly and displayed for a further 1.5 s until the next cross cue appeared.

### Performance Evaluation

Real‐time decoding results saved along with neural recordings were used to evaluate online performance. Simultaneous recordings of the participant's speech were transcribed as ground truth. Speech onset and offset times for each command were obtained using an energy‐based voice activity detection model and verified during the manual transcription process.

The chance level was calculated for real‐time usage without functional control by taking the average of n = 10000 simulations. In these chance‐level simulations, a random number generator simulated a six‐way random classification. If the simulated result was enter, the next result was corrected to back as the BCI system did in real‐time usage. These simulated results were then compared with the ground‐truth transcriptions for final chance level calculation. For functional control and offline analysis, the theoretical chance levels were used.

### Signal Stability Analysis

For the correlation analysis, the average high gamma energy of all trials (−1.0 to 1.5 s relative to speech onset) was first computed during the training data collection phase for each command. For signal stability analysis, HEG was not normalized. For each real‐time usage day, Pearson's correlation coefficient was calculated between each issued command that was decoded and their respective average training data. The average correlation of all trials was then computed for each channel. For the HGE analysis, the HGE was computed for each trial (−1.0–1.5 s) during every real‐time usage day. The average across time for all trials for each stimulus was then computed.

### Electrode Contribution Analysis

For electrode localization and cortical reconstruction, volumetric preoperative MRI and postoperative CT scans were used. They were then co‐registered using Freesurfer.^[^ ^44^ ^]^ When determining electrode contributions to decoding, the gradient of the loss function was calculated with regard to the neural activity input per trial. Subsequently, the L1‐norm of these gradients was calculated across time. Then, the average of this value was computed for all trials within each real‐time usage month to get a final score of the electrode contribution.

### Tuning Fork Control Experiments

To control for potential acoustic artifacts^[^ ^45^, ^46^ ^]^ in experiments where overt speech was attempted by the participant, a tuning fork experiment previously reported by Wilson et al. was conducted.^[^ ^47^ ^]^ A 128‐Hz tuning fork (fundamental frequency (F_0_) of the participant's voice was ≈130 Hz) was held next to the participant or gently pressed against the participant's skull. In these tuning fork experiments, no increase in energy was observed near the 128‐Hz tuning fork frequency in the ECoG channels used in this study (Figures S7 and S8, Supporting Information).

### Statistical Analysis

Performance data was either presented as raw values for each day, or in the case of ablation studies, box plots with the edges of the box representing quartiles. Data outside 1.5 times the interquartile range were shown as outliers. The maximum and minimum values of non‐outliers were shown as box plot whiskers. Each box corresponds to the accuracy for n = 33 testing days for overt real‐time experiments. Two‐sided Mann–Whitney–Wilcoxon tests with Bonferroni correction were used to assess significance. All statistical tests were performed in Python using SciPy packages.

## Conflict of Interest

The authors declare no conflict of interest.

## Author Contributions

S.L., M.A., and N.E.C. conceived and designed the experiments. S.L. designed and implemented the detection algorithm and the neural classifier. M.A., G.W.M., S.L., and C.C. implemented the real‐time decoding pipeline. C.C., S.L., and S.S. designed the user interface. S.L., M.A., and D.N.C. contributed to data collection. S.L., M.A., D.N.C., and K.WS. analyzed the data. S.L., K.WS., and B.A.W. prepared data visualizations. S.L., M.A., C.C., D.N.C., S.S., Q.R., H.H., M.S.F., and N.E.C. contributed to the study methodology. D.C.T. performed speech and language assessment. A.R.W., N.J.M., L.L.C., M.J.V., F.V.T., N.F.R. M.S.F., and N.E.C. contributed to patient recruitment and regulatory approval. W.S.A. and N.E.C. planned and performed device implantation; S.L., D.N.C., B.A.W., F.V.T., N.F.R., and M.S.F. were also involved in surgical planning and intraoperative functional mapping. H.H. supervised signal processing. N.F.R. and N.E.C. contributed to funding acquisition. N.E.C. supervised the study and the conceptualization. S.L., M.S.F., and N.E.C. prepared the manuscript. All authors reviewed and revised the manuscript.

## Supporting information

Supporting Information

Supplemental Video 1

Supplemental Video 2

Supplemental Video 3

## Data Availability

The data that support the findings of this study are available on request from the corresponding author. The data are not publicly available due to privacy or ethical restrictions.
