# Supplementary material for: Stable Decoding from a Speech BCI Enables Control for an Individual with ALS without Recalibration for 3 Months
Source: Adv Sci (Weinh). 2023 Oct 24;10(35):2304853. doi: 10.1002/advs.202304853 (PMC10724434; doi:10.1002/advs.202304853)
Supplement: Supplementary file 1 — Supporting Information [file ADVS-10-2304853-s002.pdf]

ADVANCED SCIENCE
Open Access

Supporting Information

for *Adv. Sci.*, DOI 10.1002/advs.202304853

Stable Decoding from a Speech BCI Enables Control for an Individual with ALS without Recalibration for 3 Months

*Shiyu Luo, Miguel Angrick, Christopher Coogan, Daniel N. Candrea, Kimberley Wyse-Sookoo, Samyak Shah, Qinwan Rabbani, Griffin W. Milsap, Alexander R. Weiss, William S. Anderson, Donna C. Tippett, Nicholas J. Maragakis, Lora L. Clawson, Mariska J. Vansteensel, Brock A. Wester, Francesco V. Tenore, Hynek Hermansky, Matthew S. Fifer, Nick F. Ramsey and Nathan E. Crone**

Summary of Supplementary Materials

Supplementary Videos

Supplementary Video S1 | Functional BCI control.

Supplementary Video S2 | Online communication board usage.

Supplementary Video S3 | Online communication board usage with silent speech.

Supplementary Tables

Supplementary Table S1 | Real-time session time for communication board usage.

Supplementary Table S2 | List of data collection sessions for training of the long-term speech decoder.

Supplementary Table S3 | List of data collection sessions for training of the silent speech decoder.

Supplementary Figures

Supplementary Figure S1 | Real-time decoding architecture.

Supplementary Figure S2 | Simulated long-term performance of the BCI using decoders trained with subsets of electrodes.

Supplementary Figure S3 | Confusion matrix for long-term BCI performance in each session.

Supplementary Figure S4 | Stability of the event-related high gamma activities acquired from the ECoG arrays.

Supplementary Figure S5-S6 | Event-related HGE in both training and real-time usage phase for all commands.

Supplementary Figure S7 | Power spectrogram during tuning fork experiment conducted over air.

Supplementary Figure S8 | Power spectrogram during tuning fork experiment conducted over skull.

Supplementary Figure S9 | Power spectrogram for word reading task.

Supplementary Figure S10 | User interface of the communication board application.

Supplementary Figure S11 | Simulated online performance with subsets of the electrodes in mimed experiments.

Supplementary Figure S12 | Simulated online performance in overt experiments.

Supplementary Figure S13 | Spatial activation of the ECoG grid.

Supplementary Notes

Supplementary Note S1 | Paradigms for data collection tasks.

Supplementary Note S2 | Neural decoding model.

Supplementary Note S3 | Exploratory nature of our clinical trial.

Supplementary Note S4 | Simulated online performance with the addition of lower frequency and dorsal grid information.

Supplementary Note S5 | Speech capability assessment of the participant.

References

Supplementary Videos

Supplementary Video S1 | Functional BCI control. In this video, the participant performed the following task using our BCI system: activate the functional control BCI, turn off a light, turn on the radio, turn off the radio, take control of the TV, open a video application, and select a video to watch.

Supplementary Video S2 | Online Communication board usage. In this video, the participant navigated across a 4 x 8 communication board using the BCI.

Supplementary Video S3 | Online Communication board usage with silent speech. In this video, the participant navigated across a 4 x 8 communication board using the BCI controlled by silently mimed commands.

Supplementary Tables

Supplementary Table S1 | Real-time session time for communication board usage.

Session Number	Days after implant	Session Time (min:sec)
1	194	02:57
2	194	01:46
3	196	03:07
4	196	03:23
5	197	05:15
6	210	05:43
7	211	05:33
8	215	05:22
9	217	05:02
10	218	05:19
11	222	04:08
12	224	05:00
13	225	05:13
14	229	04:43
15	231	04:31
16	232	04:48
17	235	04:52
18	236	05:15
19	243	05:13
20	245	04:10
21	246	04:29
22	250	04:44

23	252	04:54
24	253	04:23
25	257	04:39
26	260	04:26
27	264	04:45
28	266	04:53
29	267	04:59
30	271	04:53
31	273	05:03
32	278	05:09
33	280	05:15
34	281	04:59
35	285	05:27

Supplementary Table S2 | List of data collection sessions for training of the long-term speech decoder.

Days after implant	Number of sessions	Total Time (min:sec)
77	4	16:05
78	4	16:00
83	8	43:37
85	4	18:45
90	3	16:25
91	1	05:27
95	2	10:58
112	1	06:57
117	1	06:30
119	1	06:28
120	1	06:32

Supplementary Table S3 | List of data collection sessions for training of the silent speech decoder.

Days after implant	Number of sessions	Total Time (min:sec)
83	4	22:00
85	2	10:54
90	1	05:30
91	1	05:30
95	2	11:01
98	1	06:13
112	1	07:32
117	2	13:22
119	2	13:25
120	2	13:26

211	6	38:01
215	2	13:02
217	2	12:28
218	4	24:58
222	4	24:58
224	3	19:02
225	4	25:15

Supplementary Figures

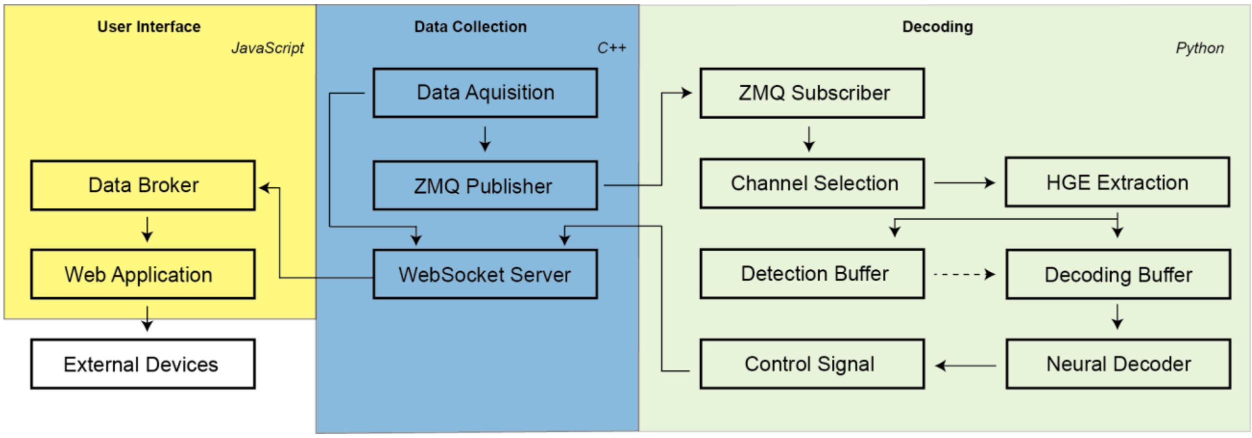

Supplementary Figure S1 | Real-time decoding architecture.

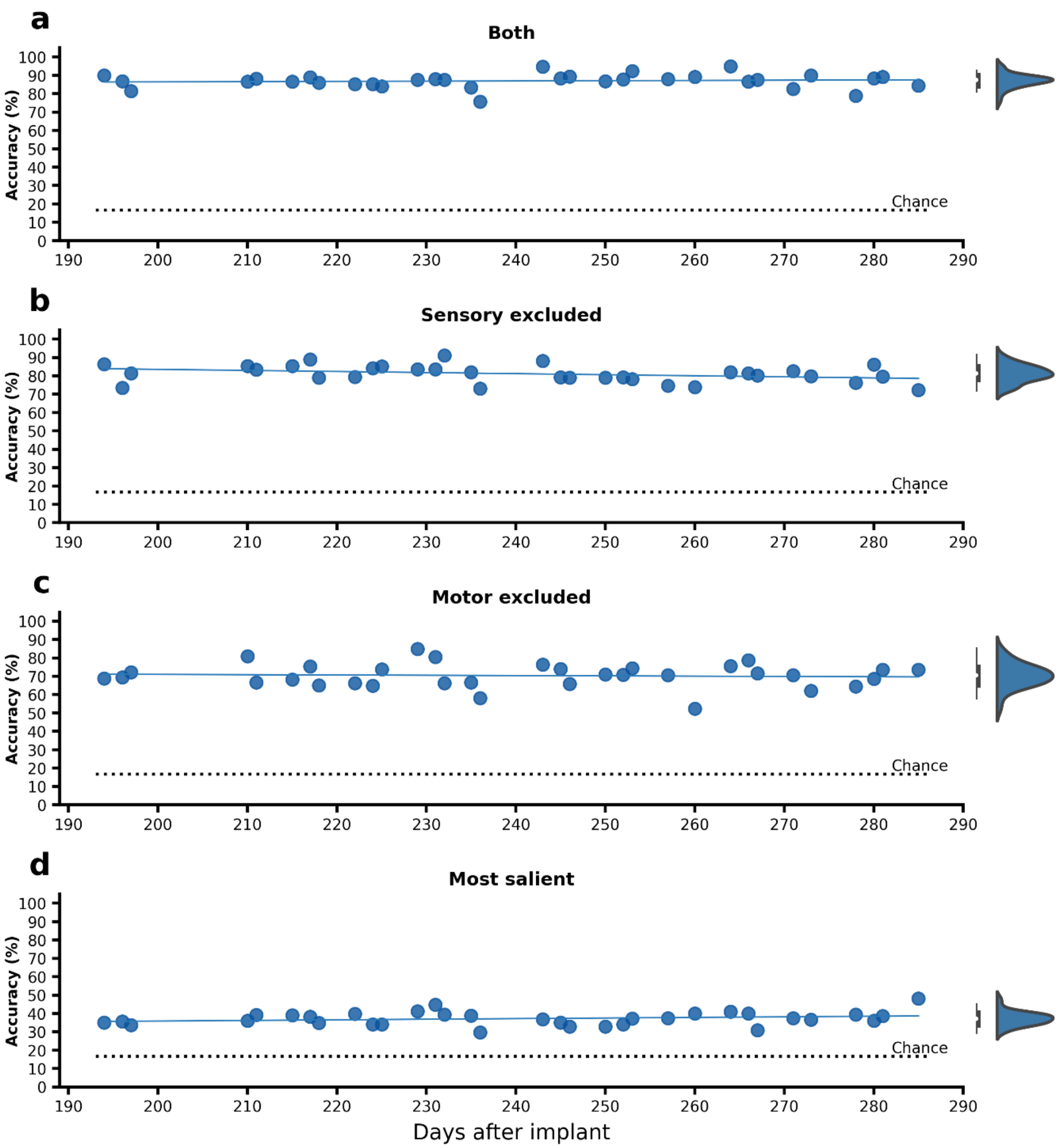

Supplementary Figure S2 | Simulated long-term performance of the BCI using decoders trained with all vs subsets of electrodes.

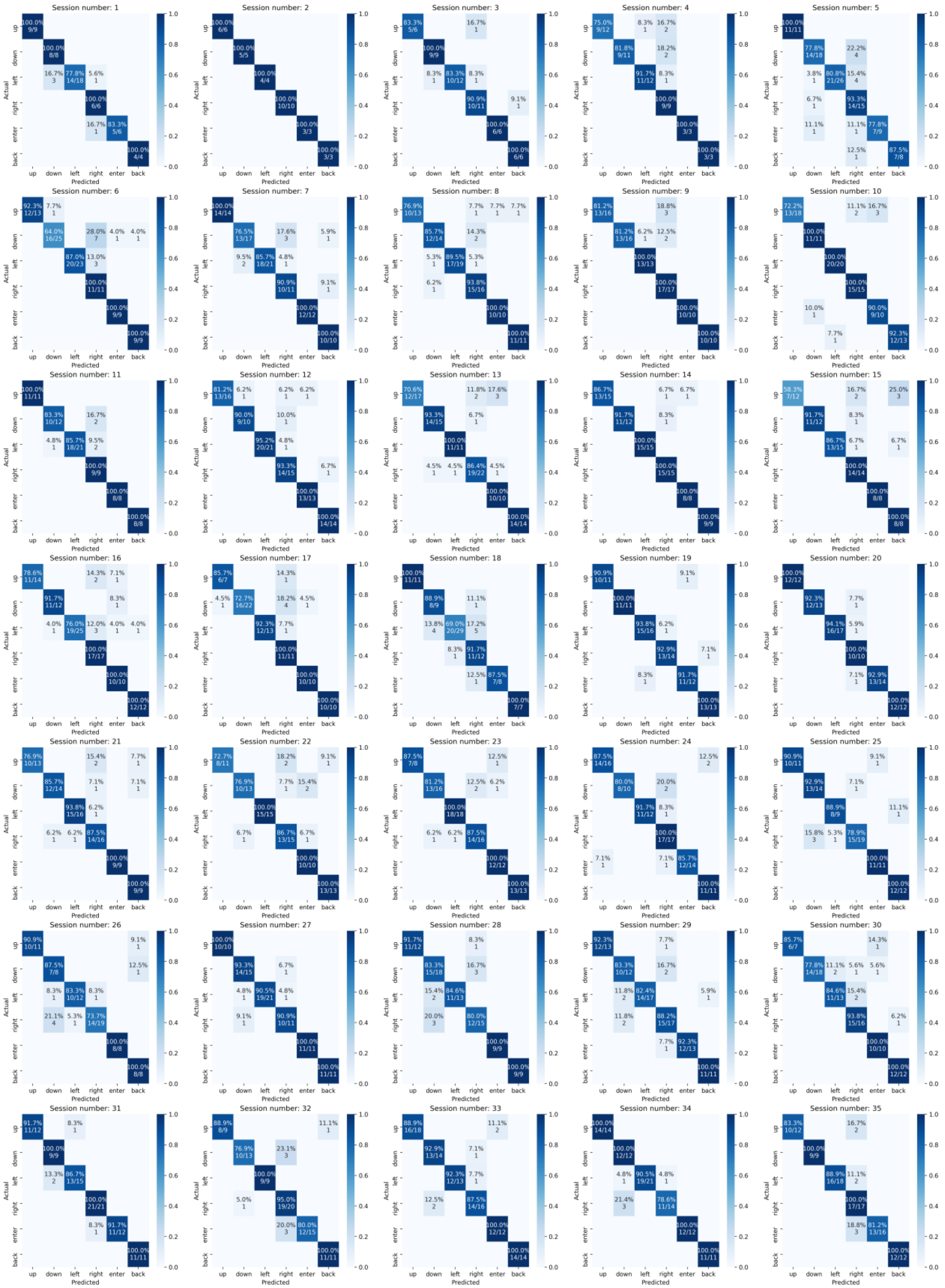

Supplementary Figure S3 | Confusion matrix for long-term BCI performance in each session.

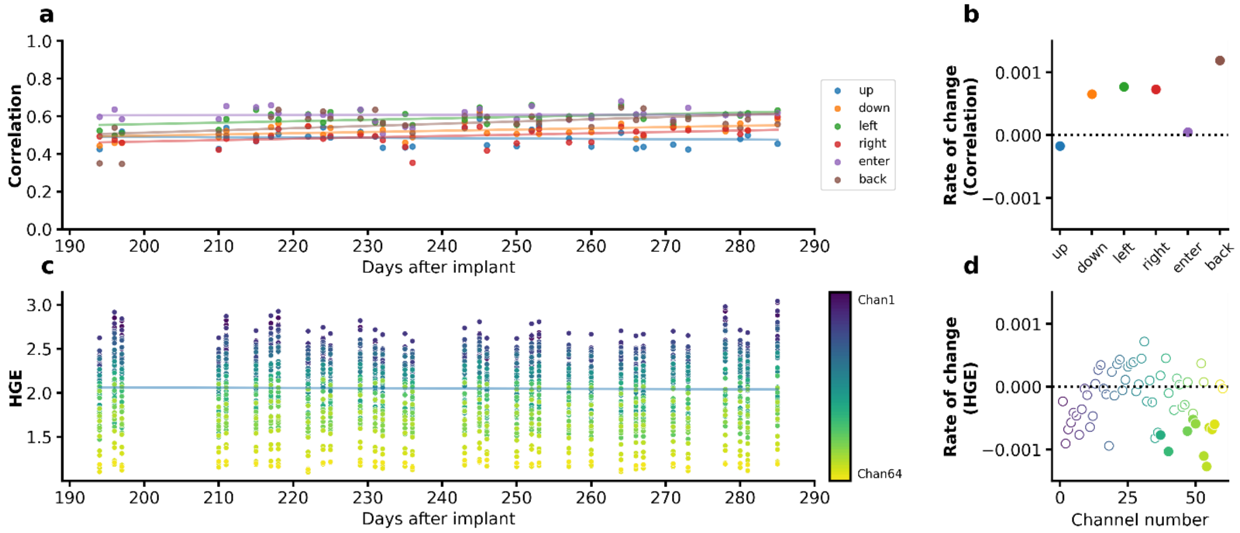

Supplementary Figure S4 | Stability of the event-related high gamma activities acquired from the ECoG arrays. a Correlation between the real-time usage trials and average training data per stimulus. For each real-time usage trial, Pearson's correlation coefficient between its HGE and the average HGE of the corresponding command in training data collection phase is calculated. Each dot represents the average correlation coefficient per usage day per stimulus. **b** Rate of change for correlations. Each dot represents one stimulus. Filled dots represent statistically significant linear relationships between correlation values and days after implant ($p < 0.05$, Wald test with t-distribution). **c** Logarithmic HGE (unnormalized) for each channel during online usage. For each day, the average HGE per channel per command was calculated. Then the final average across six commands was computed. **d** Same as b, but for HGE. Unfilled dots indicate such relationship cannot be established ($p >= 0.05$).

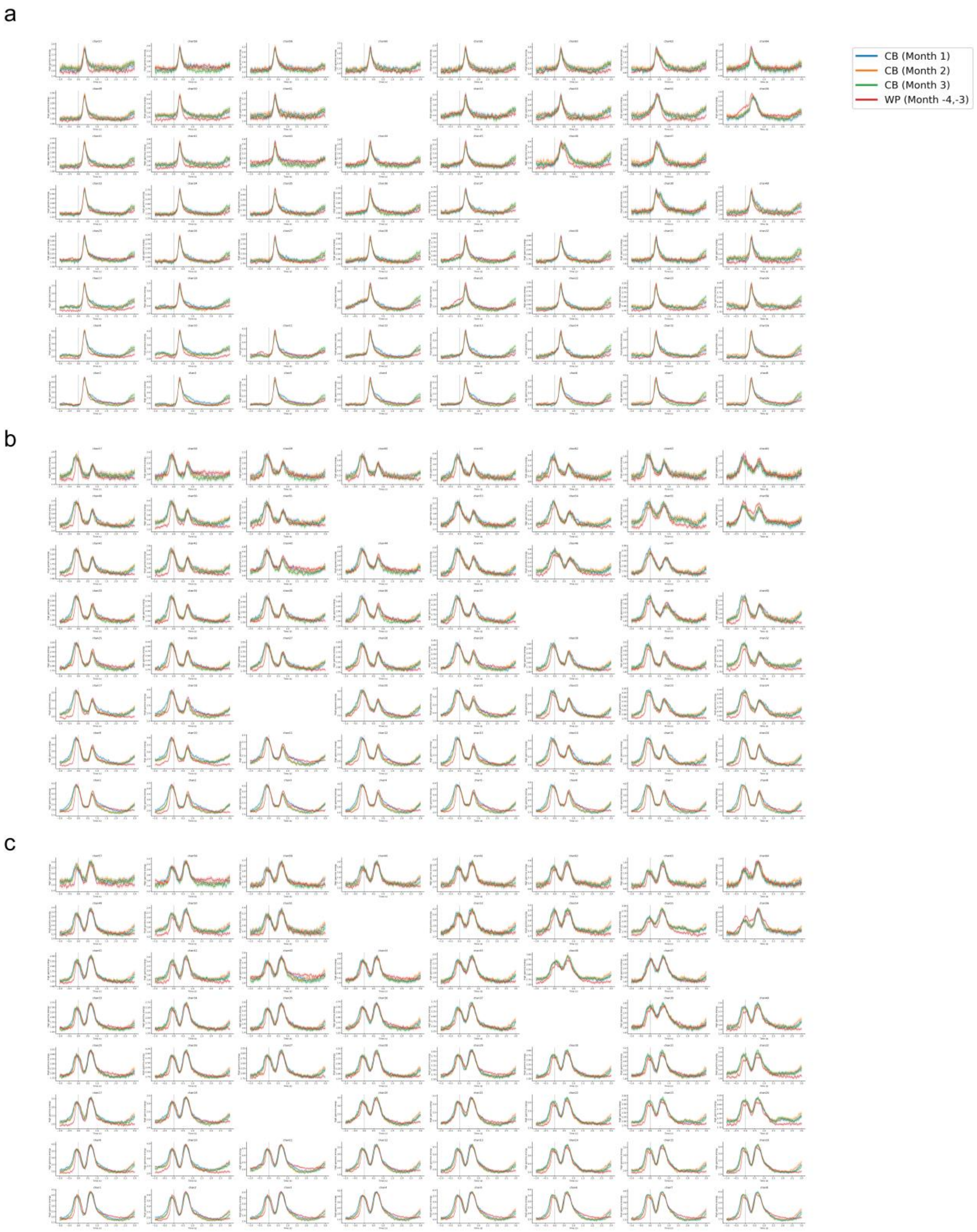

Supplementary Figure S5 | Event-related HGE in both training and real-time usage phase for all commands. a-c Up, down, and left, respectively. 0 s indicates speech onset. Shaded area represents 95% CI.

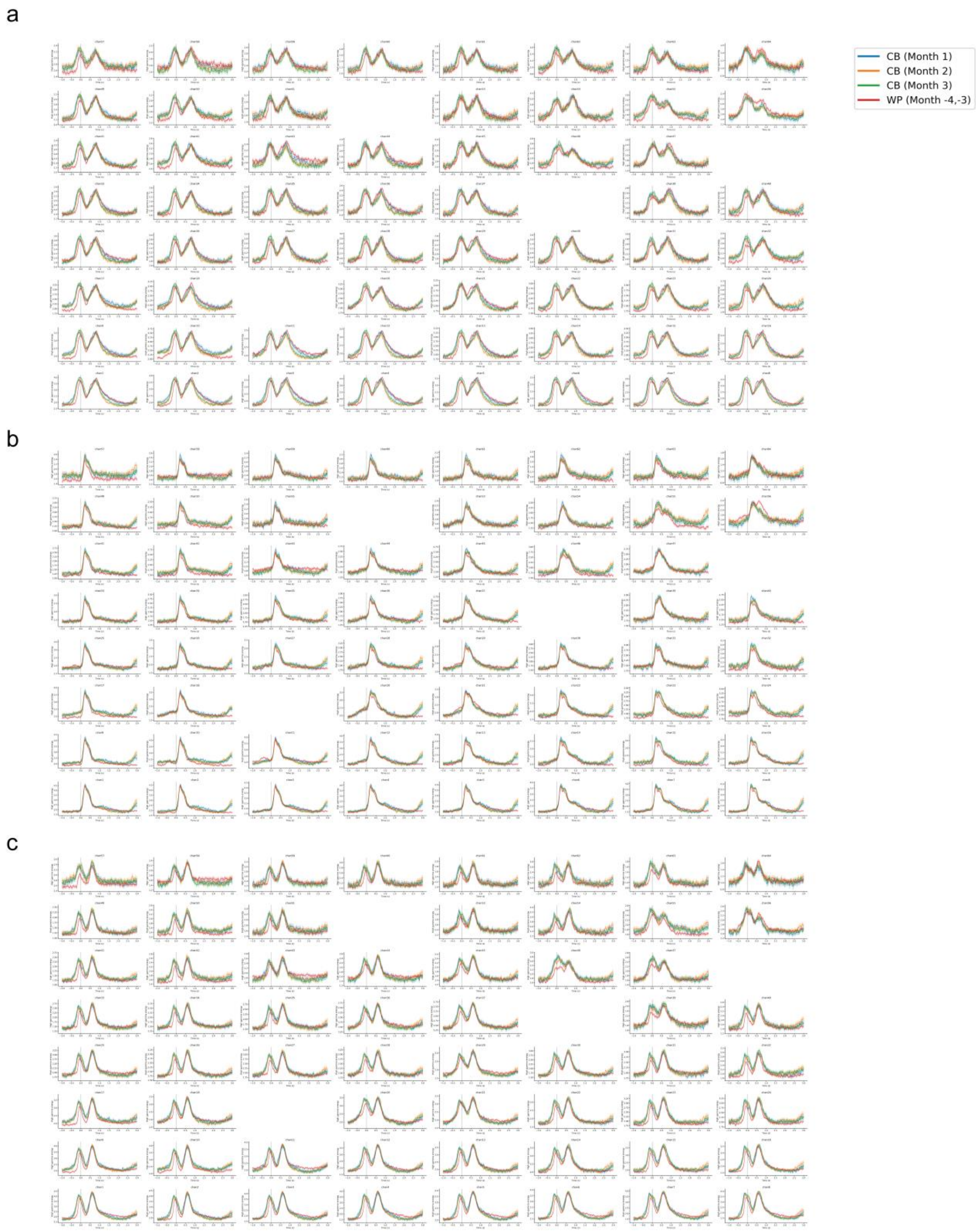

Supplementary Figure S6 | Event-related HGE in both training and real-time usage phase for all commands. a-c Right, enter, and back, respectively. 0 s indicates speech onset. Shaded area represents 95% CI.

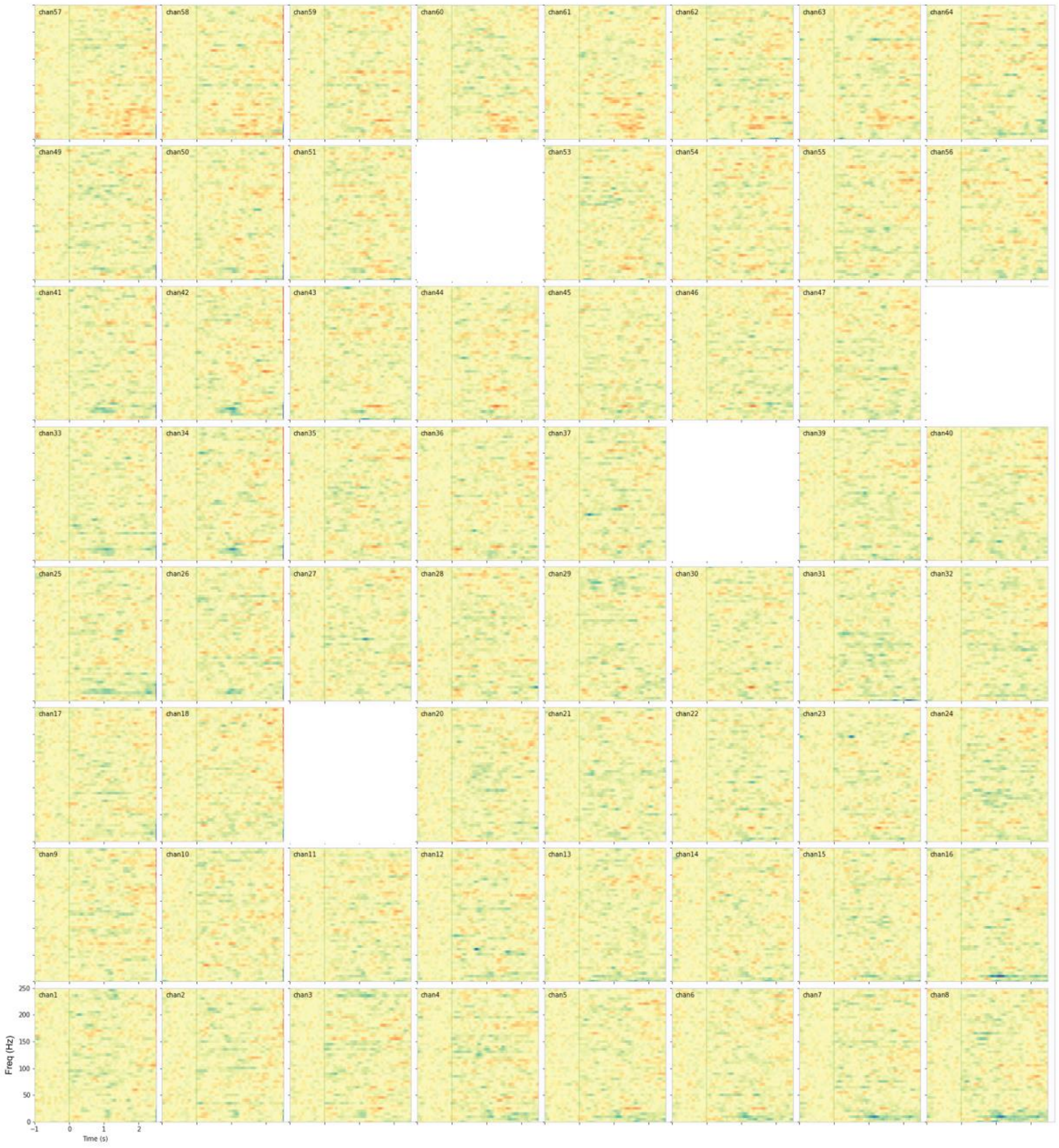

Supplementary Figure S7 | Power spectrogram during tuning fork experiment conducted over air. Average of *n*=33 trials. 0 indicates stimuli onset for the researcher to activate the tuning fork.

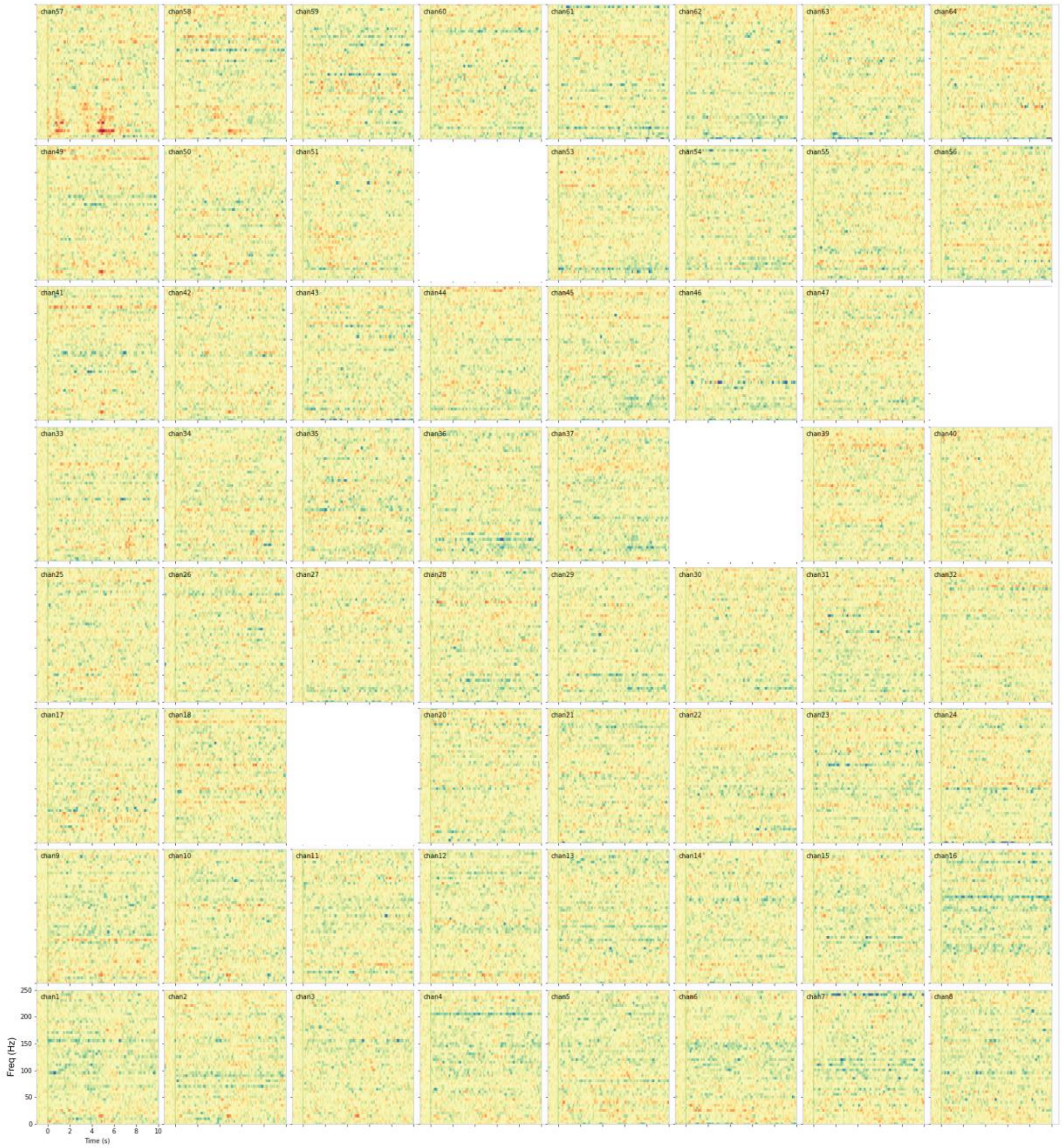

Supplementary Figure S8 | Power spectrogram during tuning fork experiment conducted over skull. Average of *n*=30 trials. 0 indicates stimuli onset for the researcher to activate the tuning fork.

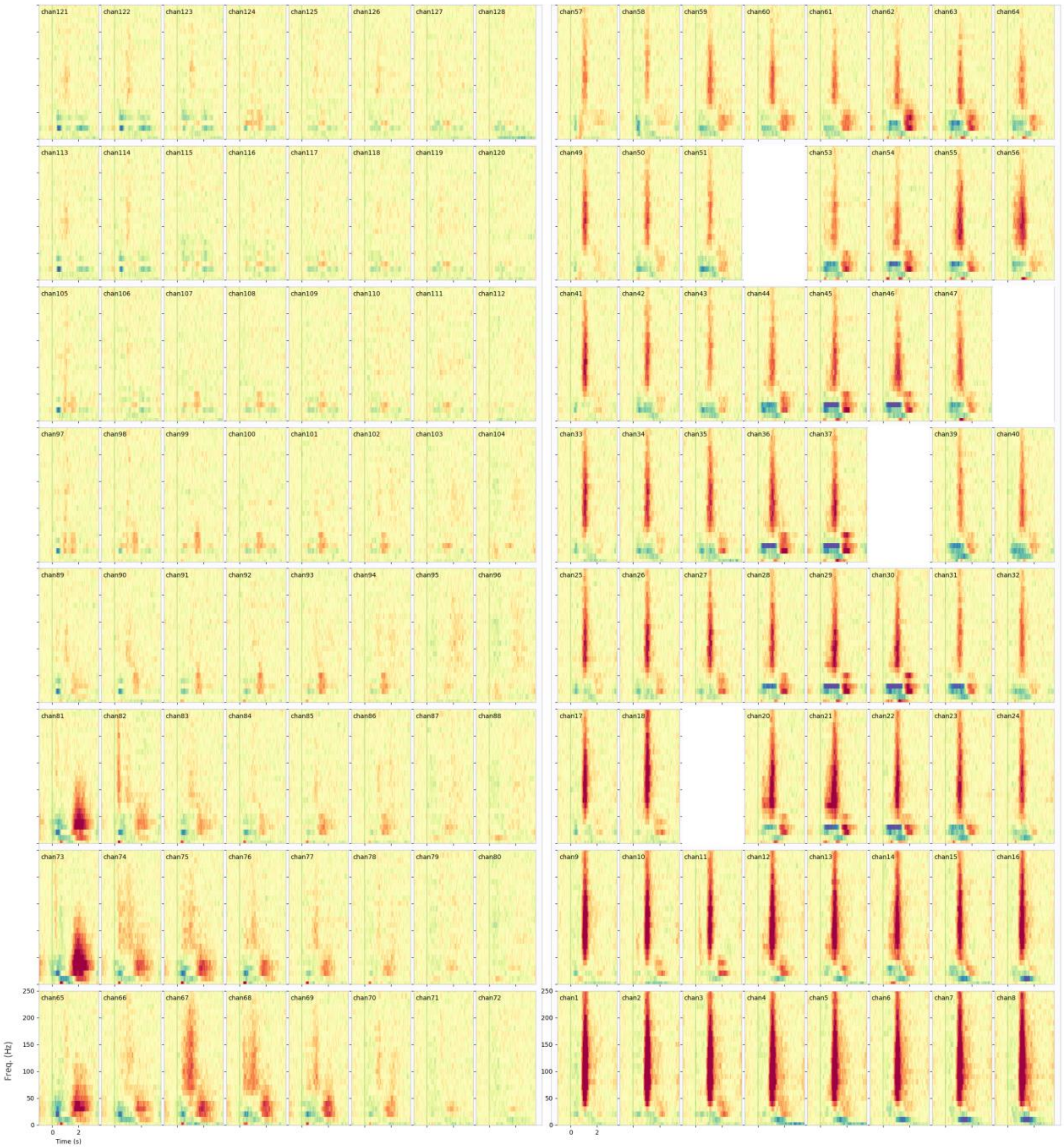

Supplementary Figure S9 | Power spectrogram for word reading task. Average of *n*=240 trials of the participant reading the word ‘up’. The left panel shows the dorsal grid while the right panel shows the ventral grid. Normalized against silence periods collected from each day. 0 indicates stimulus onset, when the word appeared on the screen.

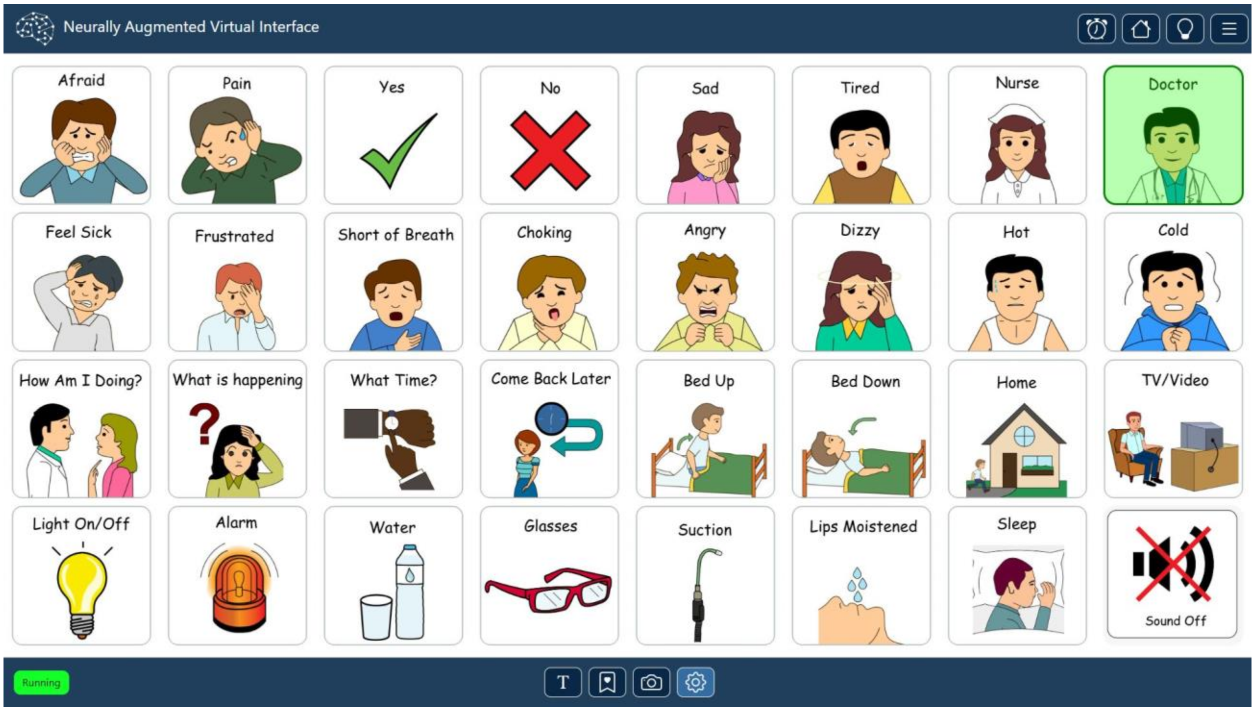

Supplementary Figure S10 | User interface of the communication board application.

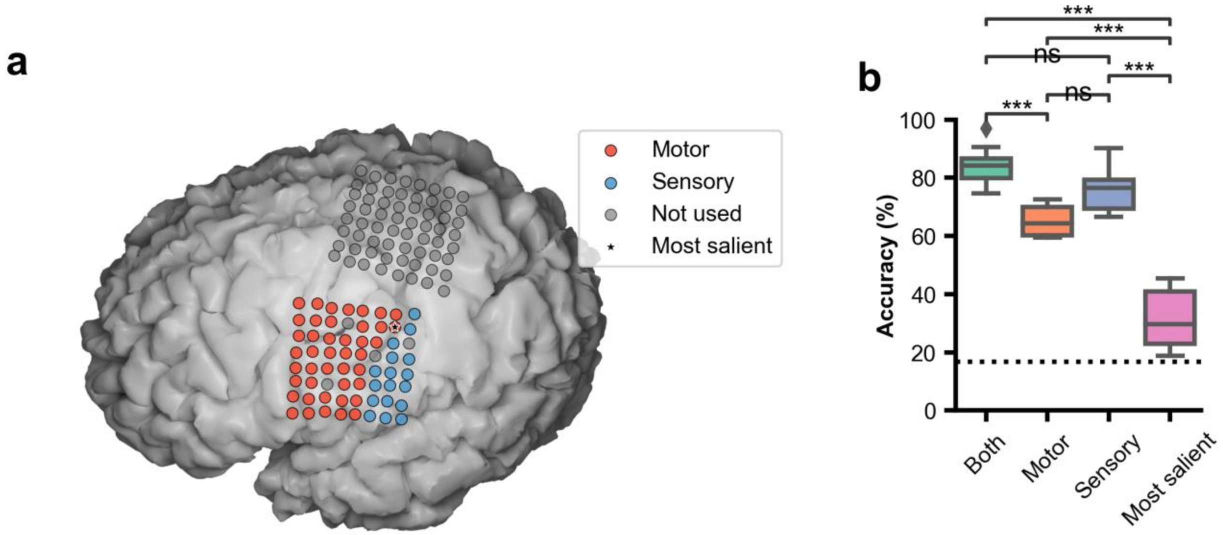

Supplementary Figure S11 | Simulated online performance with subsets of the electrodes in mimed experiments. a MRI reconstruction of the participant's brain, overlaid on top of which are the ECoG grids implanted as part of the clinical trial. Electrodes used in this study are colored in red (motor) and blue (sensory). The grey electrodes were not used in this study. **b** Simulated online accuracy when the decoding model is trained with both motor and sensory electrodes, only motor electrodes, only sensory electrodes, and only the most salient electrode. Chance = 16.67% (shown as dashed line). Each box corresponds to the accuracy for *n*=8 testing days (***p<0.001, ^{ns} Not Significant, Mann-Whitney-Wilcoxon test two-sided with Bonferroni correction).

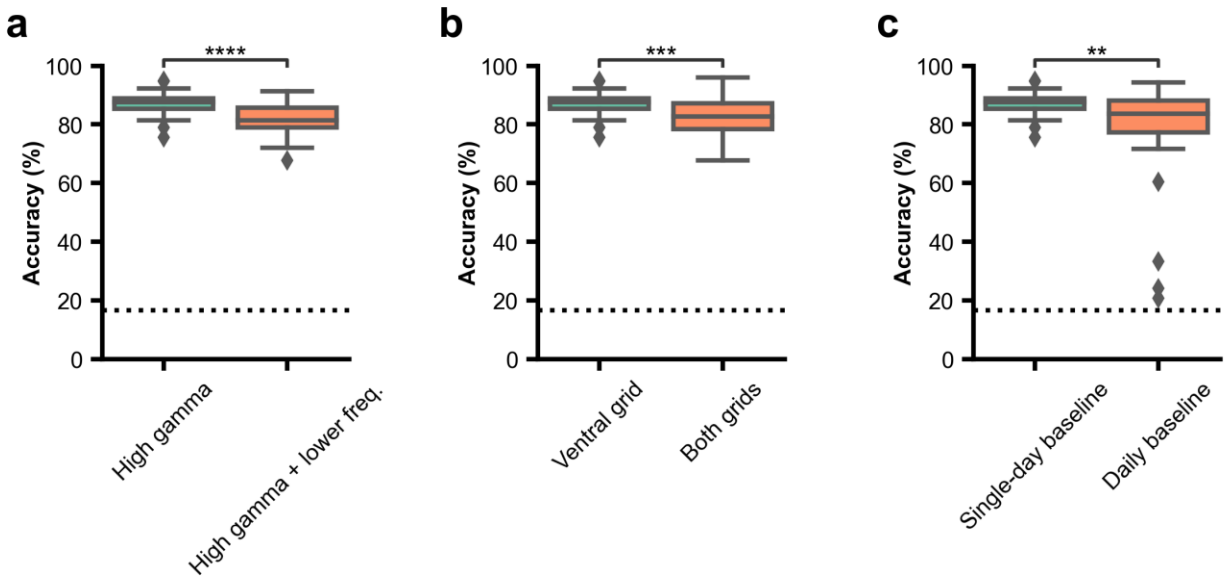

Supplementary Figure S12 | Simulated online performance in overt experiments. On the left of each panel, simulated online performance of the model trained with high gamma data from the ventral grid, normalized against baseline obtained from a single day. On the right, **a** simulated online accuracy when the decoding model was trained with high gamma and lower frequency (0.3Hz – 100Hz). **b** simulated online accuracy when the model was trained with data acquired from both the ventral and dorsal grid. **c** simulated online accuracy when the model was trained with data normalized against baseline collected on the corresponding day. Chance = 16.67% (shown as dashed line). Each box corresponds to the accuracy for *n*=33 testing days (****p<0.0001, ***p<0.001, ** p<0.01, Mann-Whitney-Wilcoxon test two-sided with Bonferroni correction).

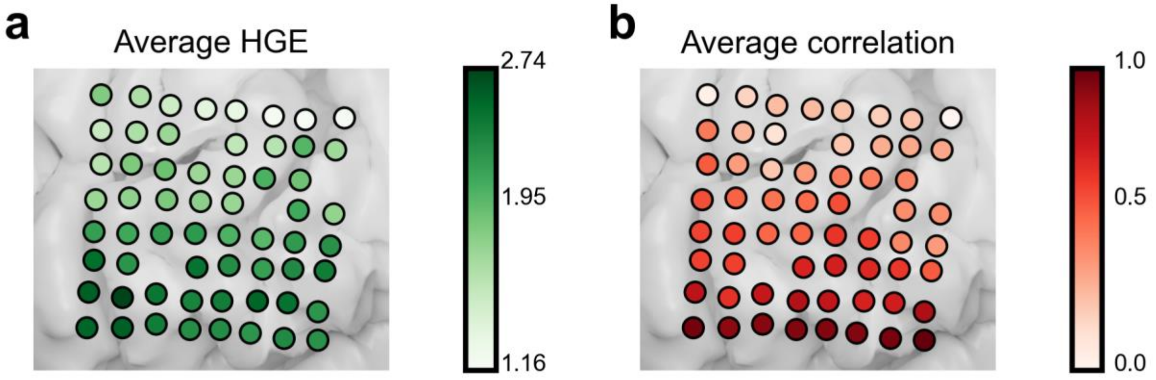

Supplementary Figure S13 | Spatial activation of the ECoG grid. a Average HGE during the study period for each electrode. **b** Average correlation between the real-time usage trials and average training data for each electrode.

Supplementary Notes

Supplementary Note S1 | Paradigms for data collection tasks.

S1.1 Word production task.

All training data for this study was collected during a word production task. The participant was instructed to speak overtly or silently single words as they appeared on a computer monitor. The stimuli used in these tasks were *up*, *down*, *left*, *right*, *enter*, and *back*. In some of the sessions, a seventh stimulus '…' was also included. The participant was instructed to stay silent upon seeing this stimulus. Each word was presented for 2 s. Interstimulus interval was 3 s.

S1.2 Syllable repetition task

Normalization statistics were collected during one single syllable repetition task. The participant was instructed to repeat overtly individual CV syllables as they were heard from desktop speakers. Each auditory stimulus lasted for 1 s. Each trial lasted between 2.5 s and 3.5 s. Exact duration was set randomly between 2.5 and 3.5 seconds with a step size of 80 ms.

Supplementary Note S2 | Neural decoding model.

As mentioned in the Methods section, we used a CNN based on InceptionTime architecture[1] for neural decoding. Our model consisted of 6 Inception[2] blocks, each with 3 Inception modules (Fig. 1d). Inside each Inception module, 3 sets of convolutions, each with 32 filters with kernel sizes ∈ {5, 11, 23}, were used after an initial convolutional layer with 32 filters of kernel size = 1. A MaxPooling layer with kernel size = 3 and one subsequent set of convolutions with 32 filters of kernel size = 1 were also used within each module. The output of these four sets of convolutions were concatenated to form the output of each module. The final output from the last Inception block was used as input to a MaxPooling layer, followed by a fully connected layer.

All training took place on desktop computers with RTX 3090 GPUs (Nvidia, Santa Clara, CA). Training samples were obtained by applying the signal processing steps and the detection algorithm on continuous data recordings from the word production tasks. During model training, we performed data augmentation by shifting the 2.5-s decoding window relative to detection onset. Let x_0 be detection onset. The following window [x_0+x_{shift}-2 s, x_0+x_{shift}+0.5 s] was used to obtain training samples, where x_{shift} was a random sample between [-0.05 s, 0.05 s). Additionally, we added gaussian noise with a relative standard deviation of 0.5 to the training samples.

Supplementary Note S3 | Exploratory nature of our clinical trial.

The clinical trial from which our results are reported is a Phase I early feasibility study to investigate the safety and preliminary efficacy of an implantable brain-computer interface. Because of the exploratory nature of our clinical trial and the limited number of trial participants, efficacy metrics, and methods to evaluate them could not be specified ahead of time and were stated as general outcomes relating to successful use of the BCI system to decode neural signals, evaluated as the accuracy and speed of decoding. Because of this, a formal statistical analysis plan was not pre-defined. Statistical analyses and interpretations thereof were informed by the actual methodology adopted and were performed to the highest statistical rigor according to relevant literature.

Supplementary Note S4 | Simulated online performance with the addition of lower frequency and dorsal grid information.

To compare the performance of our BCI with alternative methods, we retrained our decoder using the same parameters as the one used online. We added lower-frequency (bandpassed between 0.3Hz-100Hz) information and dorsal grid information to the training of two decoders, respectively.

Simulated offline performance was calculated by applying the models on neural activities acquired from online communication board experiments (*n* = 33 days). Lower accuracies were observed with addition of both the dorsal grid data (median: 82.67%, 95% CI: [80.49%, 85.39%]) and lower frequency information (median: 81.40%, 95% CI: [78.95%, 82.56%]).

Future studies are needed to verify whether the inclusion of lower frequencies or larger grid coverage would help with decoding performance in real time. The results from simulated offline experiments were limited by parameters optimized for decoding with high gamma information from the ventral grid only. In these simulated offline experiments, the model size was also kept identical as the one used in real time. We suspect at least comparale if not increased performance should be expected had the model size been incresed and hyperparameters optimized for training with the inlusion of additional information. Another factor that could have impacted the offline comparison is that neural activities used in these simulated online sessions were recorded when the participant was engaged in a closed-loop BCI experiment using the decoder trained with high gamma data from the ventral grid.

Supplementary Note S5 | Speech capability assessment of the participant.
A certified speech-language pathologist with extensive experience evaluating and treating individuals with neurogenic communication and swallowing disorders assessed speech/oral motor function. There were impairments for all functional components, including respiration, phonation, resonance, and articulation. Remarkable oral/nonspeech motor findings included weak cough on command; bilateral velar droop at rest and reduced velar elevation during phonation; lingual atrophy, limited lingual range of motion during protrusion, lateralization and elevation, decreased lingual protrusion and lateralization against resistance bilaterally; decreased labial range of movement during retraction and pucker and diminished labial seal against. Mandibular range of movement and strength against resistance were preserved. The primary perceptual speech deviation was hypernasality with nasal emission of air. Speech articulation was imprecise with all phonemes being nasalized. In addition, oral/nasal contrasts were not maintained at the word level (i.e., oral phonemes were nasalized during the production of minimal word pairs). Speech also was characterized by low volume phonation although the patient was able to increase vocal loudness on command for limited speech output. Phonatory quality was breathy and somewhat strained. There was intermittent wet-hoarse vocal quality and occasional phonation breaks. Maximum sustained phonation was 6.79 seconds for the voiced phoneme /a/; maximum sustained production of the voiceless phoneme /s/ was less than 2 seconds; both values are below the expected duration of at least 10 seconds.[3] Alternating and sequenced movements were reduced in rate (i.e., 6 repetitions of /p^/ in 5 seconds; 5 repetitions of /t^/ in 5 seconds; 7 repetitions of /k^/ in 5 seconds; 4 repetitions of "Topeka" in 5 seconds).[4] Inhalations and exhalations did not occur at appropriate linguistic junctions, resulting in short phrasing in sentences. Rate of speech was slow and labored with reading rate equal to 50 words/minute (expected rate = 150-190 words/minute).[5] Connected speech intelligibility was fair-good. In summary, the patient presented with moderate mixed spastic-flaccid dysarthria.

References

[1] H. I. Fawaz, B. Lucas, G. Forestier, C. Pelletier, D. F. Schmidt, J. Weber, G. I. Webb, L. Idoumghar, P.-A. Muller, F. Petitjean, *Data Min Knowl Disc* **2020**, *34*, 1936.

[2] C. Szegedy, W. Liu, Y. Jia, P. Sermanet, S. Reed, D. Anguelov, D. Erhan, V. Vanhoucke, A. Rabinovich, in *2015 IEEE Conference on Computer Vision and Pattern Recognition (CVPR)*, **2015**, pp. 1–9.

[3] R. D. Kent, J. F. Kent, J. C. Rosenbek, *Journal of Speech and Hearing Disorders* **1987**, *52*, 367.

[4] J. E. Pierce, S. Cotton, A. Perry, *International Journal of Language & Communication Disorders* **2013**, *48*, 257.

[5] F. Darley, D. Spriesterback, *Diagnostic Methods in Speech Pathology (2nd Ed)*, Harper And Row, New York, NY, **1978**.
